# Effect of Using Mouthwash Containing Cibotium barometz J. Smith on Cariogenic Bacteria and Acid-producing Ability of Saliva: A Randomised Blinded Clinical Trial

**DOI:** 10.3290/j.ohpd.b5574011

**Published:** 2024-07-23

**Authors:** Yu-Rin Kim, Seoul-Hee Nam

**Affiliations:** a Professor, Department of Dental Hygiene, Silla University, Busan, South Korea. Collected the data, drafted the initial manuscript, conducted the study.; b Professor, Department of Dental Hygiene, College of Health Sciences, Kangwon National University, Samcheok, South Korea. Statistical analysis, critical revisions of the manuscript, supervised the study, contributed substantially to study concept and design.

**Keywords:** biofilms, chlorhexidine, *Cibotium barometz J. Smith*, dental caries, mouthwashes

## Abstract

**Purpose:**

To examine the anti-caries effect of mouthwashes containing *Cibotium barometz J. Smith* (CB), a natural substance, and compare it with chlorhexidine and saline solution.

**Materials and Methods:**

A randomised, blinded clinical trial was conducted on 76 study participants. The differences between the 3 gargle groups (saline gargle: SAL; chlorhexidine gargle: CHX; *CB* gargle group: CB) and the differences over time (baseline, after 1 week, after 2 weeks) were compared. To this end, ANOVA was performed on caries-related clinical indicators (e.g. O’Leary plaque index, caries activity, and satisfaction).

**Results:**

The O’Leary index, caries activity, and saliva tests, gradually improved in group CB at one and two weeks. In the case of bacterial tests, unlike SAL and CHX, only in group CB did the decrease occur one and two weeks later. The caries-related indicators decreased significantly over time in group CB compared to SAL and CHX groups, and there was also a statistically significant difference in interaction between groups and time (p<0.05).

**Conclusions:**

The mouthwash containing *CB* extract showed statistically significant improvement in biofilm adhesion as well as the saliva and bacterial tests compared to SAL and CHX. However, since there were differences in the initial oral conditions of the three groups, additional long-term research is needed through crossover clinical trials to supplement these.

Dental caries is a frequent chronic disease in children and adults, and Korea’s decayed missing filled teeth (DMFT) index is still higher than that of OECD-developed countries.^39^ The World Health Organization (WHO) defines dental caries as a non-communicable disease that is not caused by infection with pathogens, is not contagious, and is related to diet and lifestyle habits.^20^ In the past, caries was considered an infectious disease caused by pathogenic bacteria.^11^ However, it is now understood that carious lesions are caused by an ecological imbalance in which acid-producing bacteria are converted into the dominant species as excessive sugar is supplied to the various microorganisms that survive in the oral cavity in the form of biofilm.^26^ In other words, it is normal for oral bacteria to be diversely composed of *Streptococcus, Actinomyces, Veillonella, Neisseria,* and *Haemophilus* species, and they are replaced by cariogenic bacteria that exist in small numbers, such as *Streptococcus mutans (S. mutans), Streptococcus sobrinus (S. sobrinus), Lactobacillus*, and *Actinomyces* species due to ecological changes in the oral environment.^10^ *S. mutans* and *Lactobacillus* species, which account for about 1–2% of the total microorganisms in the normal oral environment, rapidly increase to 55% at pH 4 after glucose is supplied, indicating that bacteria with strong acid resistance dominate and survive in a low-pH environment.^4^ In particular, *S. mutans* attaches to the acquired coating on the tooth surface and produces insoluble glucan using glucosyl transferase (GTase), allowing other bacteria to easily bind to teeth. Thus, it is necessary to suppress the production of insoluble glucan or the attachment and colony formation of acid-producing bacteria.

The use of antibacterial agents against cariogenic microorganisms has been extensively studied. For instance, chlorhexidine, which is a representative antibacterial agent, is known to be effective against root caries.^33^ However, as there is a possibility of the emergence of new resistant strains during long-term use, oral rinses containing chlorhexidine are recommended for short-term, limited use in high-caries-risk cases, due to poor oral hygiene or severe xerostomia.^36^ Antibiotic resistance occurs when microorganisms exposed to antibiotics select resistance traits during self-replication, and the resulting resistance genes are passed on to other microorganisms so they also develop the same resistance.^6^ Against this background, the ideal oral antibacterial agent is a substance that has excellent selective antibacterial activity against strains that cause oral diseases while posing a low risk to the human body. Therefore, multiple studies have tested natural substances as oral antibacterial agents.^12^ Medicinal plants occuring in nature have high pharmacological activity and low toxicity and have been used for therapeutic purposes for a very long time.^23^ In addition, plants contain various physiologically active ingredients that have antioxidant, antibacterial, and anti-inflammatory effects; thus, such plant extracts are applied in herbal medicine, pharmaceuticals, and health foods.^27^

*Cibotium barometz J. Smith (CB)* is a species of tree fern found in tropical and subtropical regions – chiefly in China, Northeast India, Malaysia, Myanmar, Indonesia, Thailand, Vietnam, and Japan – as it generally needs humid climates with sufficient sunlight.^30^ The rhizomes of *CB* are used as kidney tonics in traditional medicine and have been widely used for thousands of years for various bone diseases, such as polyuria, back pain, limb pain, sciatica, and rheumatoid arthralgia.^14^ Recent studies demonstrated that the rhizome of *CB* contains anthraquinones, flavonoids, phenolics, tannins, phytosterols, hemiterpene glycosides, and triterpenoids, which are essential in pathogen defense^32^ and also exhibit hepatoprotective activity.^28^ In addition, it affects basic alkaline phosphatase (ALP) activity and induces calcification by depositing calcium phosphate in the extracellular matrix.^19^ Several studies have already tested *CB* for cytotoxicity; its safety has been proven and guaranteed, so that *CB* is actively and widely used in various fields.^5^

Therefore, this study aimed to clinically test the efficacy of *CB* as an anti-caries agent. Specifically, this study intended to evaluate the efficacy of *CB* as an ingredient in oral gargles by comparing its effects with those of chlorhexidine and saline, which are commonly used in clinical practice, and analysing changes in bacteria and clinical indicators related to caries.

## MATERIALS AND METHODS

### Ethical Considerations

This study was conducted in accordance with the International Council for Harmonization (ICH) of Technical Requirements for Pharmaceuticals for human-use guidelines. The human study was approved by Kangwon National University (KWNUIRB-2023-01-004-001, Chuncheon, South Korea). The study was also registered as a clinical trial in the WHO International Clinical Trial Registry Platform (ICTRP) (registration date: 07/06/2023; registration number: KCT0008497; https://cris.nih.go.kr/cris/search/detailSearch.do/23815). All pertinent information (purpose, procedures, and risks) of this study was explained to all participants. Participants were free to withdraw from the study at any time. Informed consent given by all participants before being enrolled in the clinical trial.

### CB Extraction

*CB* rhizomes from Vietnam were purchased from Miryung Bio Pharm (Seoul, South Korea). After adding 70% ethanol to the crushed *CB*, conditions were maintained at 60°C for 12 h to obtain an extract. The extract was filtered using qualitative filter paper and concentrated using a rotary vacuum evaporator (N-1300E.V.S. EYELA; Tokyo, Japan). The *CB* extract was lyophilised using a freeze dryer at -80°C (Ilshin Lab; Yangju, South Korea). The powder thus obtained was -20°C after dilution.

### Study Participants 

**Fig 1:**
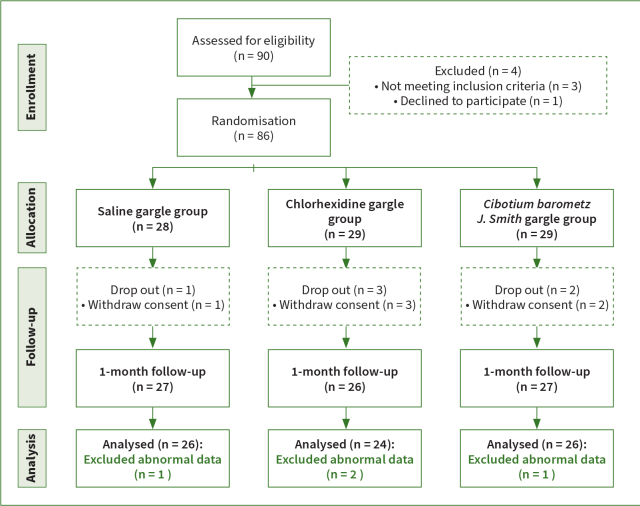
Flow chart of the study.

The sample size was calculated using the G* Power 3.1 program. The number of participants needed for the ANOVA – determined using repeated measures (within-between interaction) with statistical significance set at α = 0.05 two-tailed test, power = 0.95, effect size = 0.25, and number of groups = 3 – was 54. Ninety subjects were recruited and 86 were selected, excluding four subjects who refused to participate or did not meet the criteria. The saline gargle group (SAL) included 28 patients, the chlorhexidine gargle group (CHX) comprised 29 patients, and the *CB* gargle group (CB) had 29 patients. Excluding six subjects who did not complete the 1-month follow-up and four subjects for whom data analysis was insufficient, 76 subjects were included in the final analysis. Therefore, the final evaluation of the participants was conducted by analysing the clinical indicators of 26 SAL, 24 CHX, and 26 CB participants (Fig 1).

### Study Design and Protocol

From January 2023 to April 2023, patients who visited M Dental Clinic in Busan Metropolitan City, agreed to participate in the study and fill out the questionnaire were selected as subjects. To prevent participants from knowing to which group they belonged, the mouthwash was simply labeled “gargle solution” and all were given the same brand of toothbrush and toothpaste. Consequently, a parallel-design, randomised, blinded clinical study was conducted. Among the patients who consented to the study, 76 patients aged 20 years or older and with 16 or more remaining teeth were selected as final subjects. Subjects with one or more carious dentin lesion, diagnosed with periodontitis, who had had scaling within the previous two months, were being treated for a systemic disease that could cause bad breath or were taking antibiotics, who had serious oral diseases, or who smoked were excluded from the study.

### Clinical Examination

To ensure that the oral environment of the participants was the same, the participants visited M Dental Clinic in Busan a week before the start of the study to receive an oral examination by a dentist and undergo light scaling by two trained dental hygienists. The study began after a one-week recovery period. The maxillary right first molar (#16), maxillary left central incisor (#21), maxillary left first premolar (#24), mandibular left first molar (# 36), mandibular right central incisor (#41), and mandibular right first premolar (#44) were selected as representative teeth and clinical indices were measured. First, the baseline was set as a clinical examination one week after scaling, the O’Leary index and Cariview test were performed as caries-related indices, and the participants’ satisfaction with the natural mouthwash was confirmed. In addition, the anti-caries effect was evaluated through saliva tests, considering bacteria, acid production, buffering capacity, and caries-related bacteria.

Depending on the group (CB, CHX, SAL), participants were instructed to gargle with 15 ml oral rinse containing *CB* extract at a concentration of 10 g/ml, or 15 ml of chlorhexidine oral rinse, or 15 ml of saline oral rinse for 1 minute, respectively, three times a day (after breakfast, lunch, and dinner). Oral health management education was provided to all participants, and cariogenic foods (candy, cookies, chocolate, sugared drinks, etc.) were limited as much as possible during the study period. The patients visited the dentist periodically, i.e., at baseline (one week after scaling), one week and two weeks after using the mouthwash/ gargling. At each appointment, two dental hygienists who were trained by a dentist obtained data on caries-related indicators.

### Sociodemographic Characteristics

The sociodemographic characteristics of the study subjects were recorded. These included gender, age, marital status (married / unmarried), and presence of systemic diseases. Only subjects without systemic diseases were considered. Oral health-related factors included current dental pain, regular oral examinations, and toothbrushing instruction (TBI) experience. Lastly, satisfaction with each mouthwash was measured on a five-point scale, with higher scores indicating greater satisfaction.

### Caries-related Indicators

#### O’Leary index 

The O’Leary dental plaque test^32^ (O’Leary plaque index) was carried out. All teeth in the oral cavity were discoloured with a tooth-surface disclosing agent, and four tooth surfaces (mesial, distal, buccal, and lingual) were examined. The degree of adhesion (%) was calculated using the O’Leary index, where 1 point is given if the plaque is attached to the tooth surface and 0 points if it is not attached.^9^

#### Caries activity test

A Cariview kit (AIOBIO; Seoul, Korea) was used as a tool to evaluate caries activity according to the manufacturer’s instructions. The buccal surfaces of teeth #16 and #36 in the oral cavity were rubbed with a sterilised cotton swab and immediately placed in a culture medium. After culturing in an incubator at 37°C for 48 h, 10 drops of the indicator were added to check colour changes. Images taken using an optical analyser (Allinone Bio; Seoul, Korea) were uploaded to the manufacturer’s website and scored according to the manufacturer’s standards. According to the criteria, a Cariview score of 0.0–40.0 indicates a low risk of caries activity, and 41.0–70.0 indicates a medium risk. The range 71.0–100.0 indicates high-risk caries activity, and a low Cariview score indicates low caries activity.^35^

#### Saliva test system

Measurements were made based on the instructions of Sill-Ha ST-4910 (Arkray; Kyoto, Japan), a saliva measurement system. After rinsing the entire mouth for 10 s with the provided oral rinse, each participant was asked to spit into a sterile container, then a sample was collected using a dropper. After applying the sample to seven pads of the measuring strip, it was mounted on the measuring device’s strip holder and the cover was closed. Tooth health was automatically measured after 5 min, and results of cariogenic bacteria (10^6^ to 10^8^ colony forming units (CFU)/ml of *S. mutans* and a mixture of 3 other species [*S. mitis, S. sobrinus,* and *Lactobacillus casei*]), acid production (pH 6.0-8.0), and buffering capacity (pH 2.8–6.0) were collected. Also in terms of buffering capacity (buffering capacity was evaluated by reverse coding to ensure uniformity of variables included in the saliva tests), higher values of all variables meant poorer dental health.^22^

#### Microbiological analysis of caries

To obtain subgingival microbiota samples from periodontal pockets, a sterilised #15 paper point was inserted subgingivally at two maxillary teeth (#16 and #21) and two mandibular teeth (#36 and #41) for 10 s. The collected paper points were immediately stored in sterilised tubes and frozen at -20°C before analysis. DNA was extracted from the collected paper points using the AccuPrep Universal RNA Extraction Kit (Bioneer; Daejeon, Korea), following the manufacturer’s instructions. OligoMix (YD Global Life Science; Seongnam, Korea) and three oligonucleotides (forward primer, reverse primer, and probe) (Table 1) that react specifically to each bacterium were used.^8^ To prepare the polymerase chain reaction (PCR) reaction sample, 9 μl of OligoMix, 10 μl of 2x probe qPCR mix (Takara Bio; Shiga, Japan), and 1 μl of template DNA were combined. A 96-well plate with the PCR reaction sample was placed in the CFX96 Touch Real-Time PCR Detection System (Bio-Rad; Hercules, CA, USA) to amplify the DNA. The PCR cycle conditions were as follows: PCR initial activation step for 30 s at 95°C, denaturation for 10 s at 95°C, and annealing for 30 s at 62°C with 40 repeated cycles. The cycle threshold (Ct) parameter was calculated using the Bio-Rad CFX Manager Software program, and the number of copies was calculated by plotting the Ct value on each bacterium’s standard curve.

**Table 1:** Primers and probes used in real-time PCR assays

Bacteria	Target genes	Primers/orobe sets	Amplicon size (bp)
*Streptococcus mutans*	Mannitol-specific enzyme II (mtlA) gene	5′-CAGCGCATTCAACACAAGCA-3′ 103 5′-TGTCCCATCGTTGCTGAACC-3′ 5′-HEX-TGCGGTCGTTTTTGCTCATGG-BHQ1–3′	103
Combination of* S. mitis, S. sobrinus,* and *Lactobacillus casei*	16S ribosomal RNA	GTACAACGAGTCGCAAGCCG TACAAGGCCCGGGAACGTAT [5FAM]TAATCGCGGATCAGCACGCC[3BHQ1]	149

### Statistical Analysis

All clinical outcomes were analysed at a significance level of 5% with SPSS 24.0 for Windows (IBM; Armonk, NY, USA). One-way ANOVA and the chi-squared test were performed to determine statistically significant dfifferences by demographic characteristics among SAL, CHX, and CB. One-way ANOVA was performed to analyse clinical indicators related to caries according to group (SAL, CHX, CB) and time differences (baseline, one and two weeks later). Post-hoc analysis was performed using Duncan’s test.

The datasets used and/or analysed during the current study available from the corresponding author on reasonable request.

## RESULTS

### Sociodemographic Characteristics and Differences in Oral Health-related Factors

Table 2 shows the results regarding sociodemographic characteristics in the three groups.

**Table 2:** Characteristics of the subject in the three groups

Characteristics			N (%)		
		SAL (n = 26)	CHX (n = 24)	CB (n = 26)	p-value
*Gender	Male	14 (53.8)	12 (50.0)	12 (46.2)	0.857
	Female	12 (46.2)	12 (50.0)	14 (53.8)	
^¥^ Age (mean ± SD)		41.42±3.87	40.63±3.68	42.38±19.50	0.870
*Marriage	Single	12 (46.2)	13 (54.2)	14 (53.8)	0.810
	Married	14 (53.8)	11 (45.8)	12 (46.2)	
*Systemic disease	Absent	26 (100.0)	24 (100.0)	26 (100.0)	1.000
	Present	0 (00.0)	0 (00.0)	0 (00.0)	
*Tooth pain	No pain	26 (100.0)	24 (100.0)	23 (88.5)	0.103
	Pain	0 (00.0)	0 (00.0)	3 (11.5)	
*Regular oral examination	No regular check-ups	8 (30.8)	20 (83.3)	19 (73.1)	**<0.001**
	Regular check-ups	18 (69.2)	4 (16.7)	7 (26.9)	
*Toothbrushing instruction	No experience	4 (15.4)	18 (75.0)	16 (61.5)	**<0.001**
	Experience	22 (84.6)	6 (25.0)	10 (38.5)	

^¥^p-values were determined by one-way ANOVA, *p-values were determined by chi-squared test (p < 0.05); values are means ± standard deviations; bold: statistically significant.

### Clinical Index Based on O’Leary Plaque Index, Dental Caries Activity, and Satisfaction

Changes in caries-related indicators, including O’Leary plaque index, caries activity, and satisfaction, are presented in Table 3. Compared to baseline, the O’Leary index increased one week later in groups SAL and CHX and decreased again two weeks later. In contrast, in group CB, the O’Leary plaque index gradually and statistically significantly decreased over time. In addition, among the three groups at each time point, CB decreased statistically significantly one and two weeks later (p<0.05).

**Table 3:** Changes in the caries-related clinical indicators O’Leary plaque index, caries activity, and satisfaction for the three groups

	Variables	Estimated mean ± SE			*p-value
		Baseline	One week later	Two weeks later	
O’Leary index	SAL	55.54±2.99^aA^	71.38±0.26^aB^	68.92±0.67^aB^	**<0.001**
	CHX	50.13±4.79^aA^	61.71±4.22^bA^	59.04±4.32^bA^	0.164
	CB	59.88±2.54^aA^	54.00±2.90^bA^	46.54±2.34^cB^	**0.002**
	*p-value	0.155	<0.001	<0.001	
Caries activity	SAL	83.82±1.59^aA^	72.28±0.94^aB^	74.66±3.08^aB^	**<0.001**
	CHX	69.36±2.08^bA^	70.64±1.25^ aB^	63.75±1.68^bB^	**0.013**
	CB	73.75±2.56^bA^	69.42±2.27^aA^	61.29±1.15^bB^	**<0.001**
	*p-value	<0.001	0.445	**<0.001**	
Satisfaction with each mouthwash	SAL	1.00±0.00^aA^	1.00±0.00^aA^	1.00±0.00^aA^	**1.000**
	CHX	1.25±0.09^bA^	1.46±0.10^bA^	1.25±0.09^bA^	0.209
	CB	3.65±0.12^cA^	4.38±0.16^cB^	4.73±0.09^cB^	**<0.001**
	*p-value	**<0.001**	**<0.001**	**<0.001**	

p-values were determined by ANOVA (p<0.05); Duncan’s post-hoc test; time difference within the group are indicated by capital letters; group differences within time are indicated by lower-case letters; values are estimated means ± standard error; bold: statistically significant. The presented quantified values result from analysis and cannot be expressed in measurement units.

Caries activity decreased from baseline to one week later in SAL, but increased again two weeks later. In the CHX group, it increased from baseline to one week later, but decreased again two weeks later. However, unlike the two groups, the activity of CB gradually and statistically significantly decreased over time. In addition, comparing the differences between groups at each time point, there was no difference between groups one week later, but CB and CHX groups showed the same reduction effect two weeks later (p<0.05).

For satisfaction with the three types of gargles used, there was no statistically significant change in satisfaction scores over time in the SAL and CHX groups, but there was a statistically significant increase in satisfaction scores over time in group CB (p<0.05; Table 3).

### Saliva Test for Analysis of Cariogenic Bacteria, Acid-producing Ability, and Buffering Capacity

Table 4 specifies the results of the saliva test for caries-related indicators in the three groups. Compared to baseline, cariogenic bacteria increased one week later in the SAL group, and remained at that level until two weeks later. In the CHX group, it increased a week later compared to the baseline and decreased again after two weeks. It gradually and statistically significantly decreased in the CB group over time. Moreover, evaluating the differences between groups at each time point, no statistically significant difference was found between groups one week later, but CB and CHX groups showed the same reduction effect two weeks later (p<0.05). Acid production capacity increased over time in the SAL group. It increased one week later in the CHX groups compared to the baseline and decreased again two weeks later. In group CB, it decreased after one week compared to the baseline value and was maintained until two weeks later, but there was no statistically significant difference. In terms of the differences between the three groups at each time point, CB decreased most statistically significantly both after one and two weeks (p<0.05). Regarding buffering capacity, a higher score indicates lower buffering capacity. The buffering capacity decreased one week later in SAL compared to the baseline and increased again two weeks later. It increased one week later in CHX compared to the baseline and decreased again after two weeks. It gradually and statistically significantly decreased over time in group CB. Comparing the three groups at each time point, in group CB, buffering capacity was found to have decreased most (statistically significant difference) both after one week later and after two weeks later (p<0.05). The total value of all three variables decreased from baseline to one week later in the SAL group and increased again two weeks later. Compared to baseline, it increased one week later in the CHX group and decreased again two weeks later. The value gradually and statistically significantly decreased over time in group CB. In addition, comparing differences between the three groups at each time point, CB decreased, with the statistically most significant difference both after one week later and after two weeks later (p<0.05, Table 4).

**Table 4:** Changes in the caries-related clinical indicator saliva test according for the three groups

	Variables	Estimated mean ± SE			*p-value
		Baseline	One week later	Two weeks later	
Cariogenic bacteria	SAL	38.46±2.79^aA^	43.88±3.62^aA^	43.85±1.60^aA^	0.294
	CHX	31.00±4.04^aA^	47.04±6.24^aB^	27.88±5.17^bA^	**0.026**
	CB	50.15±3.84^bA^	41.42±3.66^aA^	27.58±4.59^bB^	**<0.001**
	*p-value	**0.001**	0.690	**0.006**	
Acid production capacity	SAL	51.35±3.12^aA^	55.54±4.56^aA^	57.38±3.82^aA^	0.531
	CHX	52.75±4.48^aA^	73.13±6.38^bB^	69.50±5.22^bB^	**0.022**
	CB	46.08±3.19^aA^	43.73±1.69^aA^	43.85±2.44^cA^	0.759
	*p-value	0.389	**<0.001**	**<0.001**	
Buffering capacity (reverse coding)	SAL	31.35±1.32^aA^	20.81±2.28^aB^	21.42±1.34^aB^	**<0.001**
	CHX	15.00±2.60^bA^	22.50±3.00^aA^	12.50±3.20^bA^	**0.050**
	CB	21.81±2.74^cA^	10.31±1.51^bB^	9.38±1.59^bB^	**<0.001**
	*p-value	**<0.001**	**<0.001**	**<0.001**	
Total	SAL	121.15±2.53^aA^	118.85±5.17^aA^	122.65±3.39^aA^	0.781
	CHX	98.75±7.15^bA^	142.67±7.87^bB^	109.88±4.92^aA^	**<0.001**
	CB	118.04±3.81^aA^	95.46±4.54^cB^	80.81±5.63^bC^	**<0.001**
	*p-value	**0.003**	**<0.001**	**<0.001**	

p-values were determined by ANOVA (p<0.05); Duncan’s post-hoc test; time difference within the group are indicated by capital letters; group differences within time are indicated by lower-case letters; values are estimated means ± standard error; bold: statistically significant. The presented quantified values result from analysis and cannot be expressed in measurement units.

### Changes in Cariogenic Bacteria in the Oral Cavity

Table 5 details the results for cariogenic bacteria as a caries-related indicator for all three groups. *S. mutans* increased one week later in SAL compared to the baseline and decreased again two weeks later. In the CHX and CB groups, the number of CFUs of *S. mutans* and the 3 other species (*S. mitis, S. sobrinus,* and *Lactobacillus casei*) gradually decreased over time, but there was a statistically significant difference only in CB. In addition, there was no statistically significant difference between the three groups at each time point, but the lowest leve of bacteria was recorded for group CM. In the SAL group, the number of CFUs of the 3 other species radually increased over time, while CHX and CB gradually decreased over time; the difference was statistically significant only in group CB. Comparing the 3 other bacterial species between groups at each time point, CHX and CB groups showed the same reduction effect both after one and after two weeks (p<0.05) (Table 5).

**Table 5:** Changes in the caries-related clinical indicators bacterial test for the three groups

	Variables	Estimated mean ± SE			*p-value
		Baseline	One week later	Two weeks later	
*S. mutans*	SAL	693.46±254.94^aA^	718.00±259.55^aA^	659.27±257.91^aA^	0.987
	CHX	1510.54±657.72^aA^	1337.42±638.48^aA^	984.29±470.20^aA^	0.816
	CB	1395.54±602.32^aA^	37.38±11.80^aB^	1.58±0.83^aB^	**0.008**
	*p-value	0.498	0.061	0.068	
*S. mitis, S. sobrinus,* and *Lactobacillus casei*	SAL	848390.88±218158.94^aA^	891257.88±236954.46^aA^	922524.69±237574.70^aA^	0.974
	CHX	465917.13±75813.94^aA^	462001.71±100596.33^abA^	386649.54±69328.15^bA^	0.749
	CB	981702.35±179995.68^aA^	271769.00±45331.84^bB^	106147.31±76917.23^bB^	**<0.001**
	*p-value	0.103	**0.016**	**<0.001**	

Values given are Ct values. p-values were determined by ANOVA (p<0.05); Duncan’s post-hoc test; time difference within the group are indicated by capital letters; group differences within time are indicated by lower-case letters; values are estimated means ± standard error; bold: statistically significant.

## DISCUSSION

Caries is an extremely common disease world-wide that occurs due to the interaction between oral microorganisms, dental plaque, and the tooth surface. Hence, research into effective treatment and prevention methods, materials, and tools is vital. Oral health problems become more severe if early treatment is missed, which is usually caused by patients’ financial constraints preventing them from receiving proper treatment.^38^

Recently, consumer preference for natural/plant-bsaed materials and medications has increased. Accordingly, the need for new anti-caries substances has emerged, and scientific interest has focused on finding effective natural products against oral pathogens. Over the past decades, the use of natural resources to develop preventive and therapeutic agents for oral health care has increased significantly.^25^^,^^34^ As natural products can be the source of new drugs, detailed screening of their bioactive effects remains a priority in developing new antibacterial agents.^16^^,^^21^ Chlorhexidine is widely used as a mouthwash and often serves as a positive control to compare the efficacy of other products.^3^ However, the use of chlorhexidine as a mouthwash is limited due to side effects, such as unpleasant taste, dryness, and burning sensation in the mouth.^24^ Therefore, this study aimed to confirm the anti-caries effect of *CB* extract as a natural alternative to chlorhexidine, which is extensively used in clinical practice. This study included 26 SAL, 24 CHX, and 26 CB subjects as a control group, with no statistically significant differences in sociodemographic characteristics between the three groups, thus ensuring homogeneity. *S. mutans* is an acid-producing bacterium and is considered a major pathogen of caries; *Streptococcus mitis (S. mitis)* and *Streptococcus sobrinus (S. sobrinus)* are also known as cariogenic bacteria.^1^^,^^31^ In particular, *Lactobacillus* species were reported to be related to progressive caries.^1^ Thus, in this study, *S. mitis, S. sobrinus,* and *Lactobacillus casei* were grouped and analysed together. Examining the bacteria collected from the subgingiva after using each gargle resulted in group CB showing a greater reduction in *S. mutans* and the 3 other species over time than in group CHX, and while group CHX exhibited a gradual bacterial reduction when used for two weeks, group CB had an immediate bacterial reduction effect when used for one week. These results are similar to a study^33^ that reported that a mouthwash containing 12 medicinally active plant species was more effective in reducing the population of *S. mutans* than chlorhexidine. Additionally, a systematic review in 2018 claimed that herbal products exert antibacterial effects against *S. mutans* similar to those of chlorhexidine.^18^ This study is very meaningful in that CB had an immediate effect compared to CHX, the effect was stronger, and it was effective not only against *S. mutans* but also against the group of combined *S. mitis, S. sobrinus,* and *Lactobacillus casei*. In addition to subgingival bacteria, the results of cariogenic bacteria contained in saliva showed that the bacterial count decreased in group CHX after two weeks of use, while CB showed an immediate decrease after a week. The efficacy of *CB* extract as a natural substance that reduces cariogenic bacteria was confirmed through an in-vitro study verifying the antibacterial effect on *S. mutans*.^7^ Although it is important to reduce the number of cariogenic bacteria due to their enamel/dentin-demineralisation effect, it is also necessary to reduce bacterial attachment to the tooth surface and plaque formation. Generally, plaque can be removed by toothbrushing, but it reattaches after 24–48 h, so it is important to reduce the reattachment rate.^29^ In this study, O’Leary plaque index showed that plaque adhesion increased compared to its state before gargling with saline solution or chlorhexidine, whereas when gargling with *CB* extract, plaque adhesion decreased over time. These results demonstrate that compared to chlorhexidine, *CB* extract reduces the reattachment of plaque, does not cause demineralisation of teeth, and thus helps maintain good dental health in the long run.

The bacteriological tests such as those mentioned here are one way to determine caries activity, but there are others. Because acid – the final metabolic product of sugar – is produced by cariogenic bacteria and is present in the saliva, the bacterial acid-producing ability and the buffering ability of saliva (which can neutralise the reduced oral pH) are also used to test caries activity.^37^ In this research, the acid-producing ability of cariogenic bacteria was confirmed through a caries activity test. The test showed when gargling with saline and chlorhexidine, the acid production ability increased, raising the risk of tooth demineralisation, whereas when gargling with *CB* extract, the acid production ability decreased in just one week. Additionally, when gargling with *CB* extract, there was an immediate effect on buffering capacity after just one week. *CB* extract showed superior effects on various clinical indicators related to caries compared to chlorhexidine and saline. *CB* activates alkaline phosphatase (ALP).^19^ ALP is an enzyme that hydrolyses organophosphate esters to locally increase the concentration of phosphate ions at the calcification site and is known to induce calcification by depositing calcium phosphate in the extracellular matrix.^17^ ALP can also improve the balance between salivary phosphate concentration and demineralisation, allowing enamel remineralisation.^2^ As ALP strengthens enamel and induces calcification by depositing calcium phosphate,^13^^,^^15^ it may affect the host’s resistance to caries. Therefore, *CB* extract, which promotes mineralisation through the activity of ALP and has antibacterial effects, can be developed into a conservative treatment for caries. Therefore, additional research on this will is needed. Meanwhile, since there are no previous studies in which *CB* has been applied in clinical practice, caution is advisable when attempting comparisons. Furthermore, the generalisability the present findings is limited since the study was limited to enamel caries. Therefore, we plan to test *CB* extract on advanced/dentin caries and conduct research on its long-term application in the future. Another limitation is that there are differences in the baseline for clinical indicators for each group. Therefore, in the future, long-term additional research will be necessary through a crossover design in which the experimental and control groups are the same to minimise errors and confounding factors.

Chemicals and alcohol contained in commercial gargles can dehydrate mucous membranes, cause irritation, and increase pain. However, as demonstrated by the satisfaction level after using each gargle for 2 weeks, satisfaction in the CB group was very high at 4.73 points, compared to 1.25 points in the CHX group. Therefore, it can be assumed that satisfaction with and efficacy of this natural substance as an anti-caries oral rinse have been proven. Since the extract has been confirmed to have potential as an anti-caries agent, it can be actively used to prevent and arrest caries. In addition, it is suggested that the natural *CB* extract has high potential as an alternative anti-caries gargle to widely-used chlorhexidine.

## CONCLUSION

The use of mouthwash containing *CB* extract showed a positive effect on clinical indicators related to caries. Over time, the reattachment of plaque and the acid-producing ability of cariogenic bacteria were reduced. In addition, satisfaction with the buffering capacity of saliva and using gargles was high, indicating an evident decrease in cariogenic bacteria in the oral cavity. Therefore, *CB* extract can be considered a natural agent effective in clinical indicators related to caries, and it can help maintain an optimal oral environment and contribute to improving oral health.
